# Healthcare use in the year preceding a diagnosis of pancreatic cancer: a register-based cohort study in Denmark

**DOI:** 10.1080/02813432.2022.2069730

**Published:** 2022-04-29

**Authors:** Linda A. Rasmussen, Line F. Virgilsen, Claus W. Fristrup, Peter Vedsted, Henry Jensen

**Affiliations:** a Research Unit for General Practice, Aarhus, Denmark; b Department of Surgery, Odense Pancreas Centre (OPAC), Odense University Hospital, Odense, Denmark; c Danish Pancreatic Cancer Database, Odense, Denmark

**Keywords:** Pancreatic neoplasms, early detection of cancer, general practice, health services research, Denmark

## Abstract

**Objective:**

To describe the use of healthcare prior to a diagnosis of pancreatic cancer in Denmark.

**Design:**

A population-based cohort study using prospectively recorded data from Danish National Health Registries.

**Setting:**

Danish general practice and hospitals.

**Subjects:**

A total of 5926 patients diagnosed with pancreatic cancer in 2012–2018 and 59,260 matched references without pancreatic cancer from the Danish general population.

**Main outcome measures:**

The monthly frequency of healthcare use (contacts and tests in general practice and contacts and diagnostic investigations in hospitals) during the 12 months preceding the pancreatic cancer diagnosis and a corresponding index date assigned to the references.

**Results:**

Compared to the references, the patients had increased contacts and diagnostic tests, especially blood glucose testing, in general practice from 7 to 12 months before diagnosis. Hospital contacts and diagnostic imaging increased from 5 months before the diagnosis.

**Conclusions:**

The pattern of increasing healthcare contacts before a diagnosis of pancreatic cancer may represent a window of opportunity to diagnose pancreatic cancer earlier. The increased use of blood glucose test in general practice may represent an important sign of an underlying disease.

**Key points**
- Pancreatic cancer is a rapidly progressing and highly lethal disease. Focus on early diagnosis is essential to improve the prognosis.
- Patients with pancreatic cancer had increased number of healthcare contacts from 7 months before the diagnosis.
- Patients with pancreatic cancer had increased number of blood glucose tests taken throughout almost the entire year before the diagnosis.
- The results may indicate that a window of opportunity exists to diagnose pancreatic cancer earlier.

## Introduction

Pancreatic cancer is a rapidly progressing and highly lethal disease, which is reflected in a 5-year survival rate of less than 5% [1]. Approximately 80% of pancreatic cancer patients in Denmark are diagnosed with locally advanced and metastatic cancer, which, combined with a frail population, aggressive tumours and limited treatment options, is the primary cause of the dismal prognosis [1,2]. Prompt referral for diagnostic workup and earlier diagnosis in less advanced stages may facilitate curative surgery and improve the prognosis in patients with pancreatic cancer [1].

Little evidence exists on the diagnostic pathway for patients with pancreatic cancer, and existing studies are based mainly on small-scale, single-site trials in countries with other healthcare systems than the Danish system [2–4]. As no effective screening methods or biomarkers are currently available [5], symptomatic presentation is key to initiate a diagnostic evaluation. Yet, the diagnosis of pancreatic cancer is difficult due to vague, unspecific symptoms in the early stages and low predictive values of ‘a priori’ signs and symptoms [2,4,6,7].

General practice is the place of first presentation for 70–75% of patients with pancreatic cancer in the UK and France, and 85% in Denmark [3,4,8]. A recent Danish study found an increase in abdominal imaging from up to six months before a pancreatic cancer diagnosis [9], and it is well established that cancer patients have higher healthcare use from 6 to 12 months before the cancer is diagnosed [10,11]. This can be seen as a proxy for symptom presentation and the actions taken by healthcare professionals [10–12]. However, no studies have explored when and how patients seek healthcare in the period leading up to a diagnosis of pancreatic cancer.

The overall aim of this study was to establish knowledge of the diagnostic pathway for patients with pancreatic cancer in Denmark by analysing the patients’ healthcare use in the year preceding a diagnosis of pancreatic cancer.

## Materials and methods

### Study design and setting

We conducted a register-based study and linked data at the individual level through the unique personal registration number assigned to all Danish citizens at birth or immigration [13]. Tax-funded healthcare is offered to all citizens in Denmark free of charge. General practice serves as gatekeeper to the rest of the healthcare system, and more than 98% of the Danish population is registered with a specific general practice [14].

### Population

All persons in Denmark diagnosed in the period from 2012 to 2018 with a first-time diagnosis of pancreatic cancer recorded in the Danish Pancreatic Cancer Database (DPCD) and the Danish Cancer Registry (DCR) were eligible for inclusion. Pancreatic cancer was defined in accordance with the International Classification of Diseases, 10th revision (ICD-10) for malignant neoplasms of pancreas as C25 (except endocrine pancreas, ICD-10: C25.4) and including cancer in the papilla Vateri, ICD-10: C24.1.

Ten references without a history of pancreatic cancer matched on sex, age and general practice were identified in the Civil Registration System [13] through incidence density sampling. An index date was assigned, corresponding to the diagnosis date of their matched cancer case.

### Data sources

Data were obtained from six national population-based registers. The DPCD [15] is a clinical database with prospective registration of diagnostic workup, diagnosis, treatment and outcomes of patients with pancreatic cancer in Denmark since 2011. The completeness of the database has increased from 72% in 2011 [15] to 100% in 2017 [16]. The DPCD provided information on pancreatic cancer diagnosis codes and diagnosis dates, tumour stage and pancreatic cancer treatment. If the pathological tumour stage was available, this registration was used. Otherwise, and in the non-resected patients, the clinical tumour stage registered in the DPCD was used. The DCR [17] supplemented with information on pancreatic cancer diagnosis codes, diagnosis dates and tumour stage if missing in the DPCD. The Danish National Patient Register [18] provided information on all somatic inpatient and outpatient visits, including diagnoses, diagnostic investigations and tumour stage if missing in the DPCD and the DCR. Information on healthcare contacts in general practice was obtained from the Danish National Health Service Register [19]. Finally, the Danish Civil Registration System [13] provided information on age and sex, and Statistics Denmark [20] provided demographic and socioeconomic information.

### Outcomes

Outcomes were mean monthly rates of healthcare activity in the 12 months preceding the diagnosis or index date. The following measures of healthcare activity were analysed: (1) number of contacts in general practice (daytime face-to-face consultations, telephone consultations and email consultations), (2) number of hospital contacts (admissions and outpatient visits); overall and restricted to specialities involved in the pancreatic cancer diagnosis (general surgery and surgical gastroenterology) and restricted to medical specialities, (3) number of diagnostic investigations in general practice (blood glucose tests, urine dipstick tests and haemoglobin measurements), diagnostic imaging in hospitals (X-ray, magnetic resonance imaging (MRI) scan, computed tomography (CT) scan and ultrasound) and diagnostic imaging used for targeted diagnosis of pancreatic cancer (abdominal ultrasound, thoracic/abdominal/pelvic MRI scan and CT scan).

### Other variables

Sex, age, marital status, educational level, disposable income, ethnicity and comorbidity were used to account for possible confounding. Age, marital status and educational level were measured at the diagnosis or index date. Marital status was categorised into ‘cohabiting’ and ‘living alone’. The highest attained educational level was categorised according to UNESCO’s International Standard Classification of Education [21] into ‘basic’ (≤10 years), ‘short’ (11–15 years) and ‘long’ (>15 years). The patient’s income was based on household income from the calendar year before the index date, calculated according to the OECD-modified scale [22] and categorised into tertiles defined as ‘low’, ‘middle’ and ‘high’ income. Ethnicity was categorised into ‘Danish, including descendants of immigrants’ and ‘immigrant’. Comorbidity burden was defined according to Charlson’s Comorbidity Index (CCI) [23] on the basis of diagnosis codes from hospital contacts registered in the Danish National Patient Register during the 10-year period before the study entry. CCI scores were categorised into ‘low’ (score 0), ‘moderate’ (scores 1–2) and ‘severe’ (scores ≥3).

Pancreatic cancer stage and pancreatic cancer resection were used to describe the progression of the cancer. Stage was defined according to the classification system by the Union for International Cancer Control (UICC) and categorised into ‘localised’ (stage I + II), ‘regional’ (stage III), ‘distant’ (stage IV), ‘unknown’ and ‘missing data’. Pancreatic cancer surgery was categorised into ‘resection with curative intent’ and ‘palliative surgery or no surgery’.

### Statistical analyses

Differences in population characteristics between patients with pancreatic cancer and the reference population were investigated by Pearson’s Chi-square test. A negative binominal regression model applying cluster robust variance estimation at the patient level was used to calculate incidence rate ratios (IRRs) with confidence intervals (CIs) for comparison of the monthly rates of healthcare activity between pancreatic cancer patients and their references. The IRRs were adjusted for age, marital status, educational level, disposable income, ethnicity and comorbidity. Unadjusted rates and adjusted IRRs were presented graphically. Changes in rates were assessed visually and identified as the last month before rates changed compared to the month after.

All analyses were stratified for sex as gender differences are known to exist in healthcare utilisation [24]. Furthermore, four sub-analyses were performed; contacts to general practice and hospitals were stratified on type of pancreatic resection and on metastatic and non-metastatic disease (excluding ‘unknown’ and ‘missing’). Furthermore, outcome measures for patients with metastatic disease were compared to the corresponding figures for patients with non-metastatic disease, and finally, number of blood glucose tests performed in general practice were analysed stratified on pancreatic cancer patients with and without diabetes at study entry.

## Results

The study included 5926 patients with pancreatic cancer and 59,260 references. The median age was 70 years (interquartile interval: 64–77), and 48% were women. Patients with pancreatic cancer had more comorbidity and slightly lower educational level and disposable income compared to the reference population (Table 1).

**Table 1.** Study population characteristics of pancreatic cancer patients diagnosed in 2012–2018 and references without pancreatic cancer^a^.

	Pancreatic cancer patients	Reference population
	*N* (%)	*N* (%)
Sex		
Male	3103 (52.4)	31,030 (52.4)
Female	2823 (47.6)	28,230 (47.6)
Age at index date		
Median, IQI^b^	70 (64; 77)	70 (64; 77)
Age groups (years)		
18–49	189 (3.2)	1905 (3.2)
50–64	1412 (23.8)	14,068 (23.7)
65–74	2405 (40.6)	24,037 (40.6)
75–84	1575 (26.6)	15,832 (26.7)
85+	345 (5.8)	3418 (5.8)
Educational level		
Basic	**2286** (**38.6)**	**22,052** (**37.2)**
Short	**2692** (**45.4)**	**26,327** (**44.4)**
Long	**948** (**16.0)**	**10,881** (**18.4)**
Disposable income		
Low	**2073** (**35.0)**	**18,873** (**31.8)**
Middle	**1986** (**33.5)**	**19,544** (**33.0)**
High	**1867** (**31.5)**	**20,843** (**35.2)**
Marital status		
Cohabitant	**3447** (**58.2)**	**36,402** (**61.4)**
Living alone	**2479** (**41.8)**	**22,858** (**38.6)**
Country of origin		
Danish	5627 (95.0)	56,067 (94.6)
Immigrant	299 (5.0)	3193 (5.4)
Comorbidity^c^		
Low	**3541** (**59.8)**	**38,129** (**64.3)**
Moderate	**1675** (**28.3)**	**15,645** (**26.4)**
Severe	**710** (**12.0)**	**5486** (**9.3)**
Diabetes^e^		
Yes	**836** (**11.1)**	**5137** (**6.8)**
Tumour stage		
Localised	633 (10.7)	n/a^e^
Regional	1031 (17.4)	n/a
Distant	3176 (53.6)	n/a
Unknown	108 (1.8)	n/a
Missing data	978 (16.5)	n/a
Surgery		
Curative resection	1335 (22.5)	n/a
Palliative surgery	329 (5.6)	n/a
No surgery	4262 (71.9)	n/a

Significant differences between groups are shown in bold (Pearson’s chi-square test).

^a^ The references were matched 1:10 on sex, age and general practice. Matching on age was based on a maximum deviation of two years compared to the pancreatic cancer patients.

^b^ IQI: interquartile interval.

^c^ Charlson’s Comorbidity Index score defined at study entry: low = score 0, moderate = scores 1–2, severe = scores ≥3.

^d^ Defined at study entry.

^e^ n/a: not applicable.

### Contacts to general practice

**Figure 1.**
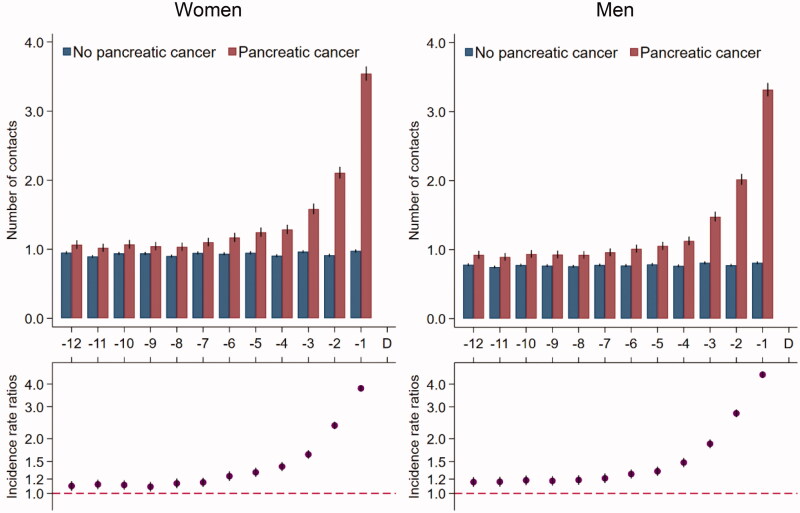
Contacts* in general practice for 5926 pancreatic cancer patients and 59,260 references without pancreatic cancer. *Number of contacts include daytime face-to-face contacts, email consultations and telephone consultations in the 12 months prior to the pancreatic cancer diagnosis date and a corresponding index date assigned to references without pancreatic cancer. Number of contacts are presented as crude rates of mean number of contacts per month (upper part) and incidence rate ratios (lower part) adjusted for age, marital status, ethnicity, educational level, household income and comorbidity. Black lines represent 95% confidence intervals.

**Figure 2.**
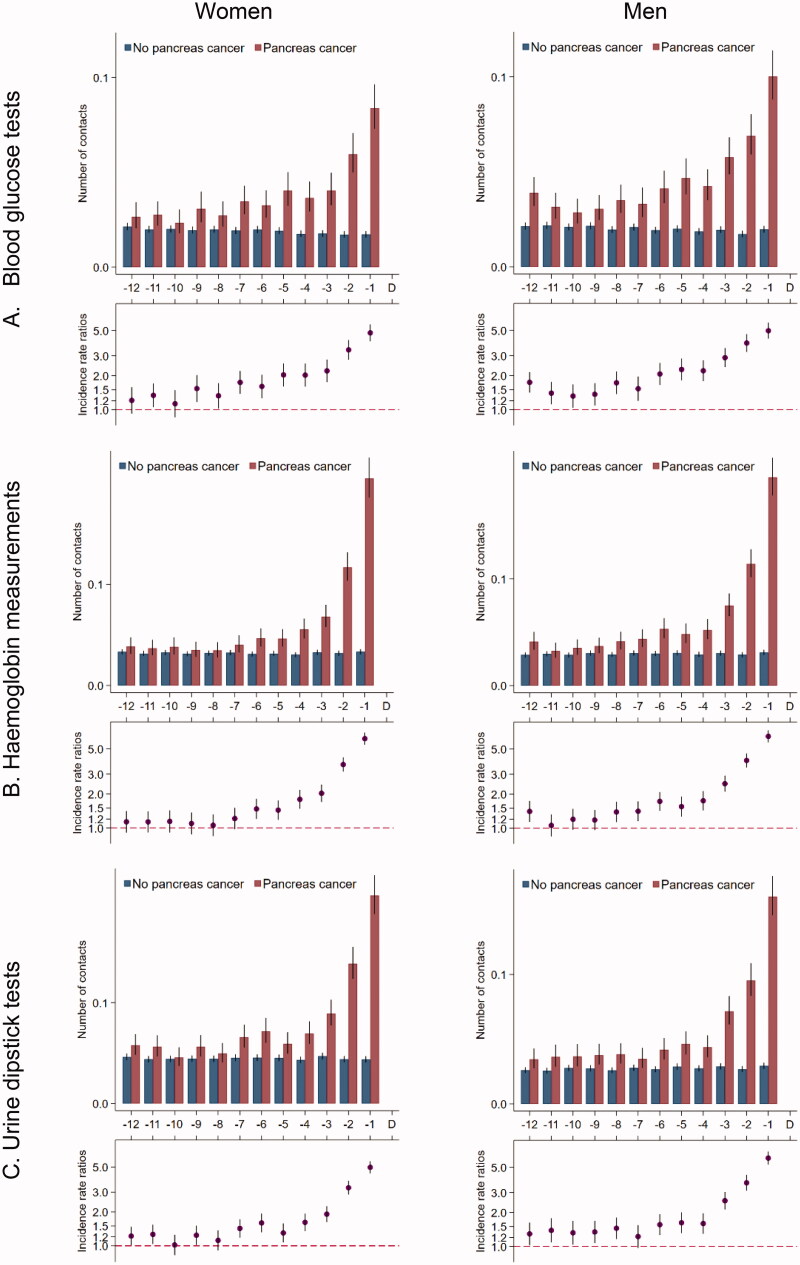
Tests* in general practice for 5926 pancreatic cancer patients and 59,260 references without pancreatic cancer. *Number of tests in the 12 months prior to the pancreatic cancer diagnosis date and a corresponding index date assigned to references without pancreatic cancer. Number of tests are presented as crude rates of mean number of tests per month (upper part) and incidence rate ratios (lower part) adjusted for age, marital status, ethnicity, educational level, household income and comorbidity. Black lines represent 95% confidence intervals.

Women with pancreatic cancer had an average of one contact per month in general practice from 12 months until seven months prior to the diagnosis (Figure 1); this number increased from seven months before the diagnosis to an average of 3.5 contacts in the last month before diagnosis. Compared to their references, women with pancreatic cancer had statistically significantly more contacts during all 12 months before the index date; this figure increased to 3.8 times more contacts in the last month before the index date (IRR: 3.78 (95% CI: 3.66–3.92)). Results were similar in men and for tests in general practice (Figure 2).

### Contacts to hospitals

**Figure 3.**
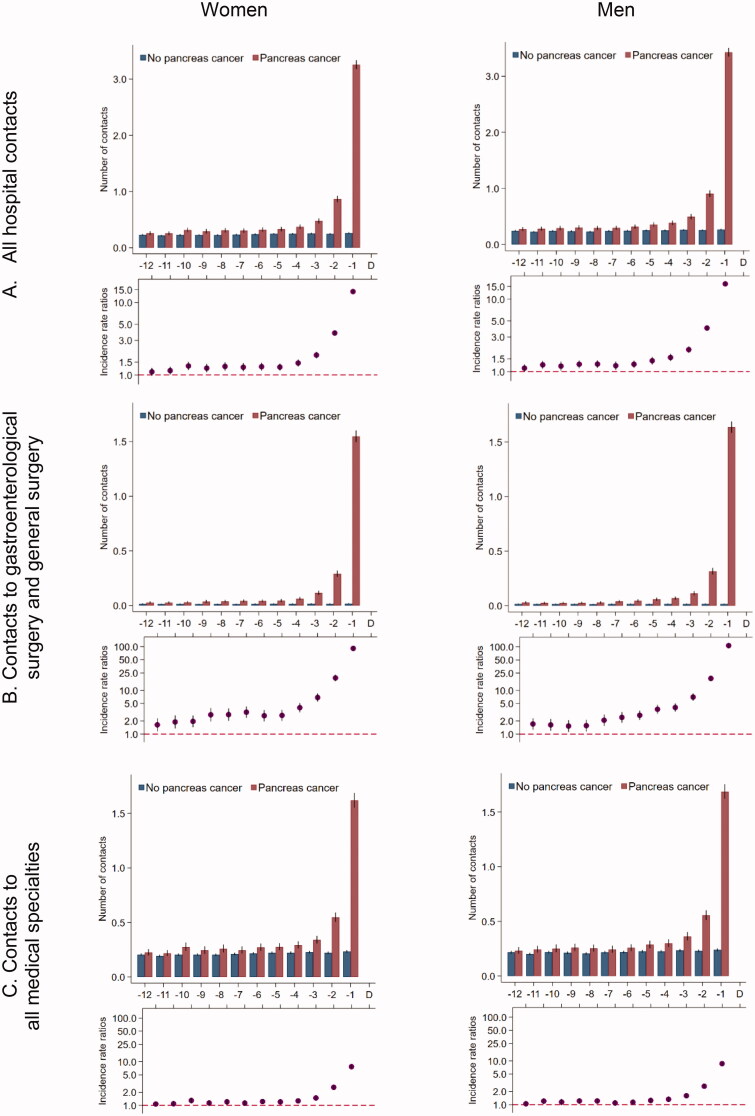
Number of hospital contacts* for 5926 pancreatic cancer patients and 59,260 references without pancreatic cancer. *Number of contacts in the 12 months prior to the pancreatic cancer diagnosis date and a corresponding index date assigned to references without pancreatic cancer. Number of contacts are presented as crude rates of mean number of tests per month (upper part) and incidence rate ratios (lower part) adjusted for age, marital status, ethnicity, educational level, household income and comorbidity. Black lines represent 95% confidence intervals.

Patients with pancreatic cancer had statistically significantly more hospital contacts from 11 months prior to the diagnosis date compared to references and increased rates from five months before the diagnosis date (Figure 3(A)). When we restricted to hospital specialities involved in the diagnosis of pancreatic cancer, the adjusted contact rates were statistically significantly higher in pancreatic cancer patients throughout the entire study period compared to their references (Figure 3(B)). For medical hospital contacts, the adjusted contact rates increased in the last three to four months (Figure 3(C)).

### Diagnostic imaging

**Figure 4.**
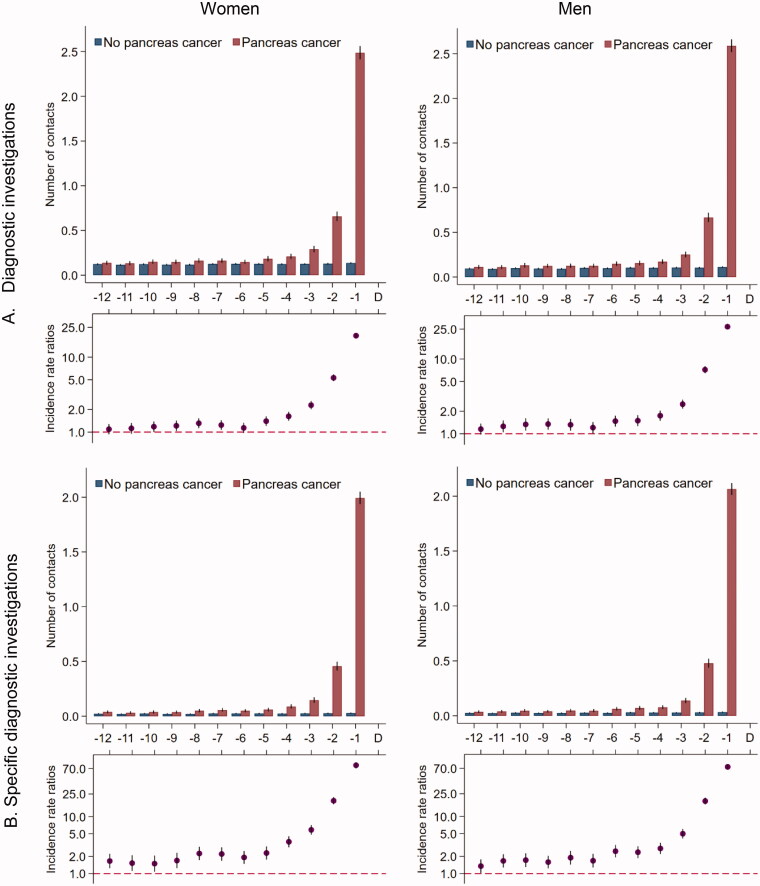
Number of diagnostic investigations* for 5926 pancreatic cancer patients and 59,260 references without pancreatic cancer. (A) All MR scans, CT scans, ultrasound and X-ray. (B) Ultrasound abdomen, MR thorax/abdomen/pelvis, CT thorax/abdomen/pelvis. *Number of diagnostic investigations in the 12 months prior to the pancreatic cancer diagnosis date and a corresponding index date assigned to references without pancreatic cancer. Number of diagnostic investigations are presented as crude rates of mean number of investigations per month (upper part) and incidence rate ratios (lower part) adjusted for age, marital status, ethnicity, educational level, household income and comorbidity in model A and comorbidity in model B. Black lines represent 95% confidence intervals.

The overall number of diagnostic imaging examinations performed in patients with pancreatic cancer increased slightly from five months before the diagnosis date and peaked in the last two months (Figure 4(A)). Patients with pancreatic cancer had statistically significantly more diagnostic investigations performed that were directly relevant for pancreatic cancer throughout the entire study period, and the numbers increased in the last five to six months before the diagnosis (Figure 4(B)).

### Sub-analyses

Analyses on healthcare contacts stratified on patients with and without metastatic disease compared to their references resembled the ones in the main analyses in both groups, and findings were similarly stratified into patients undergoing curative intent surgery and patients undergoing palliative or no surgery (data not shown). Analyses on healthcare contacts in patients with metastatic disease compared to patients without metastatic disease showed no statistically significantly differences, except for more contacts in general practice in the last month before the diagnosis in patients with metastatic disease (Supplementary material Figure 1A). Further, more hospital contacts or diagnostic investigations performed were seen from three months before the diagnosis in patients without metastatic disease, however, with a reverse pattern in the last month except in specific hospital contacts (Supplementary material Figure 1B, 1C, 1E and 1F). When restricting the analyses to pancreatic cancer patients without diabetes and their references, the increased number of blood glucose tests remained statistically significantly increased in the pancreatic cancer patients compared to their reference from eight to nine months before the diagnosis (Supplementary material Figure 2).

## Discussion

### Statements of principal findings

We found statistically significantly more contacts and diagnostic tests in general practice throughout the last year before the diagnosis among patients with pancreatic cancer, which increased further in the last seven months before diagnosis compared with matched references. For contacts and blood glucose tests, this increase was seen throughout the entire year before the diagnosis and when restricting the analyses to pancreatic cancer patients without diabetes and their references, the increased number of blood glucose tests remained statistically significantly increased in the pancreatic cancer patients compared to their reference from eight to nine months before the diagnosis. The number of hospital contacts and diagnostic imaging increased from five months before the diagnosis, i.e. several months later than in general practice. Contact rates to hospital specialities related to pancreas cancer were higher all 12 months and increased further from nine to 10 months before the diagnosis.

### Strengths and weaknesses of the study

An important strength of the study was the population-based design and the use of nationwide registers with high validity and completeness [13,15,17–20] combined with the free access to healthcare in Denmark. This reduced the risk of bias and increased the generalisability of the results to the total population of patients with pancreatic cancer in Denmark. Furthermore, the large study population provided high statistical precision, and the study gained strength from the information on diagnosis date and cancer treatment reported in the DPCD.

The indication for tests performed in general practice was unknown, and the increased number of blood glucose tests in patients with pancreatic cancer could be caused by more patients with diabetes in this population compared to the reference population. However, the increase in blood glucose tests was also apparent in patients without diabetes from eight to nine months before diagnosis. Another limitation was the lack of information on the results of performed blood glucose tests. However, the pattern in the number of performed blood glucose tests indicates that this measure served as a relevant proxy for fluctuating blood glucose levels, which are likely to occur in connection with new onset of diabetes originating from the development of pancreatic cancer. Thus, this measure may serve as a proxy for healthcare activity related to symptoms of pancreatic cancer.

The statistically significantly more contacts and blood glucose tests in general practice at study entry among patients with pancreatic cancer compared to references may suggest that the increase set in before study entry. Thus, we analysed this healthcare use for 24 months before the diagnosis, and found stable rates and IRR from 24 to 8 months before the diagnosis. This increased healthcare use at baseline might be caused by residual confounding from comorbidity. Comorbidity scores were based on diagnosis codes from hospital records; however, comorbidity diagnosed and handled solely in general practice might not be registered in hospital records. Such under-registration might cause overestimated IRRs, as pancreatic cancer patients had more comorbidity compared to the matched population (see Table 1).

### Findings in relation to other studies

The pattern in healthcare activity in general practice supported previous findings that general practice is involved in the diagnostic trajectory for a majority of cancer patients [25]. Previous studies have reported similar patterns of increased contacts to general practice from seven months before a cancer diagnosis [10–12,26]. Monthly face-to-face contact rates in general practice in the 12 months before a pancreatic cancer diagnosis increased from 0.5 to 1.8 (data not shown). Studies on colorectal cancer [27] and intracranial cancer [10] showed lower monthly face-to-face contact rates in the 12 months before the diagnosis, increasing from 0.4 to 1.4. These studies were conducted on the same data sources and using the same methodology. This indicates more contacts to general practice in patients with pancreatic cancer, which is in line with a study on 19 cancer types by Lacey et al. [28], who found that patients with pancreatic cancer were most likely to have visited their GP at least three times before referral.

The present findings of increases in diagnostic investigations and hospital contacts are similar to previous studies [10–12] on healthcare use prior to a cancer diagnosis. Further, a recent Danish study on 11 abdominal cancer types demonstrated increased rates of abdominal ultrasound and CT scans from three to five months before a pancreatic cancer diagnosis [9]. Singh et al. [29] found that it was difficult to identify pancreatic cancer through imaging and that 80% of pancreatic tumours were missed on a CT scan prior to the diagnosis. Furthermore, several scans are often required to ensure sufficient imaging quality for a precise diagnosis and evaluation of cancer stage, even after a suspicion of pancreas cancer has been raised. This may explain part of our findings that patients with pancreatic cancer underwent more specialised diagnostic imaging throughout the entire study period, especially in the last months before the diagnosis, compared to their references.

### Interpretation and implications for the future

The results indicate that general practice is highly involved in the diagnosis of pancreatic cancer. The onset of increasing contacts and diagnostic tests is seen several months earlier in general practice than in hospitals, which suggests that GPs may have potential to expedite the diagnosis of pancreatic cancer. However, the vague and unspecific symptoms of pancreatic cancer remain a challenge as they have low predictive value in the early stages, which makes it difficult to use this opportunity for earlier diagnosis [6]. The increase in blood glucose testing from seven months before the pancreatic cancer diagnosis indicates that the GP suspects diabetes over pancreatic cancer. The increase was seen in both pancreatic cancer patients with and without a diagnosis of diabetes at study entry, indicating that it may be seen as a proxy for new symptom presentation in patients with known diabetes. It is a difficult task for GPs to balance the risk of a later cancer diagnosis against over-investigation and causing worries in patients who are unlikely to have cancer [7]. Reluctance to overburden hospital systems or cause unnecessary anxiety may leave the patients presenting with no alarm symptoms of cancer with poor safety netting [30]. GPs may need to raise their level of suspicion for symptoms that could suggest certain cancer types [28], and the study underlines the importance of considering and investigating for cancer even when the patient does not present with well-known alarm symptoms [25]. Thus, access to relevant diagnostic investigations seems to be an important tool to prevent delay in the diagnostic pathway.

No firm conclusions could be drawn from the results as to why some patients are diagnosed in non-advanced stages and when curative intent surgery is still an option. The timing of the onset of increased healthcare contacts was similar for patients diagnosed with and without metastatic disease (Additional file 1, Figure 1) and in patients undergoing curative intent surgery and palliative or no surgery. This may suggest that the large proportion of patients diagnosed in advanced stages, ineligible for curative surgery, is not caused by further delay in the diagnostic trajectory. However, this should be investigated further before any conclusions can be made.

The higher number of hospital contacts and diagnostic investigations from four to five months before the diagnosis in patients without metastatic disease compared to patients with metastatic disease (Supplementary material Figure 1) may be explained by the need for more complex and time-demanding diagnostic evaluation to prepare for surgery. Furthermore, a diagnostic delay related to the registration of the diagnosis may explain the earlier onset of the steep increase in contacts from patients without metastatic disease compared to patients with metastatic disease; the diagnosis date is registered at the date of surgery in patients undergoing surgery, and waiting times for surgery could be up to three to four weeks in the studied period.

## Conclusions

Patients with pancreatic cancer had increased numbers of contacts and diagnostic tests in general practice from seven months before the diagnosis and increased hospital contacts and diagnostic imaging starting four to five months before the diagnosis. General practice and specialised hospital departments involved in diagnosing pancreatic cancer may use this window of opportunity to diagnose pancreatic cancer earlier. Diagnosing a rapidly developing pancreatic cancer, which is indicated only by vague and unspecific symptoms in the early stages, is a challenging task. The presented findings call for high levels of safety netting, better access for GPs to relevant diagnostic investigations and urgent referral for fast-track diagnostic pathways, and efficient cross-sectoral cooperation.

## Supplementary Material

Supplemental Material
