# Supplementary material for: Healthcare use in the year preceding a diagnosis of pancreatic cancer: a register-based cohort study in Denmark
Source: Scand J Prim Health Care. 2022 Apr 29;40(2):197–207. doi: 10.1080/02813432.2022.2069730 (PMC9397460; doi:10.1080/02813432.2022.2069730)
Supplement: Supplemental Material [file IPRI_A_2069730_SM6618.pdf]

Appendix Figure 1: Contacts and tests* for 3,176 pancreatic cancer patients with metastatic disease (red bars) and 1,664 references with local/regional disease (blue bars).

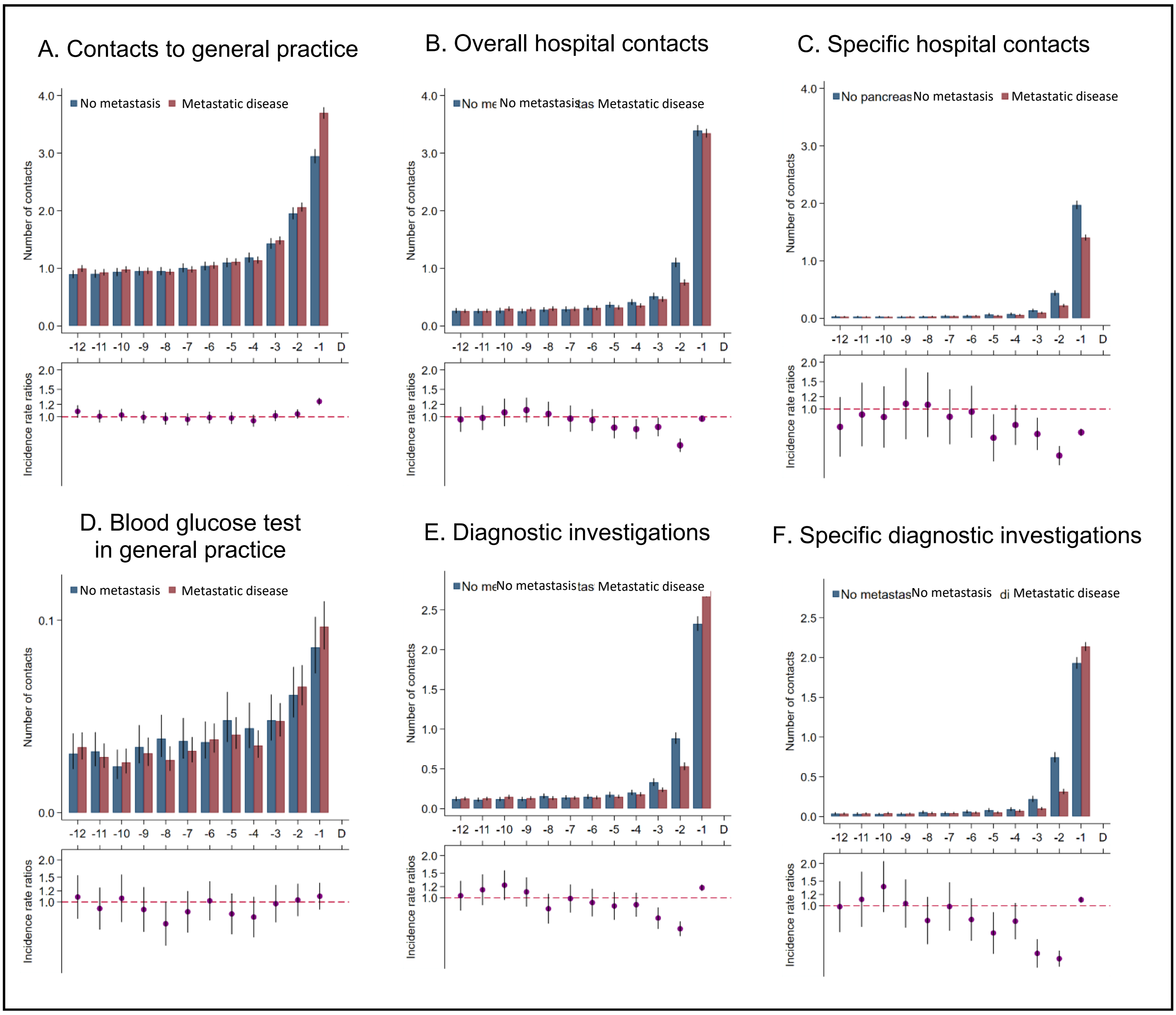

C: General surgical and surgical gastro-enterological departments.
E: All MR scans, CT scans, ultrasounds and X-rays
F: Ultrasound abdomen, MR thorax, abdomen, pelvis, CT thorax, abdomen, pelvis

* Number of contacts/tests in the 12 months prior to the pancreatic cancer diagnosis date. Number of contacts/tests are presented as crude rates of mean number of contacts/tests per month (upper part) and incidence rate ratios (lower part) adjusted for sex, age, marital status, ethnicity, educational level, household income and comorbidity in model A, B, D and E, and sex and comorbidity in model C and F. Black lines represent 95% confidence intervals.

Appendix Figure 2: Number of blood glucose tests* performed in general practice for pancreatic cancer patients with diabetes (n=836) and without diabetes (n=3,176), and 8,360 and 31,760 sex- and age-matched references without pancreatic cancer

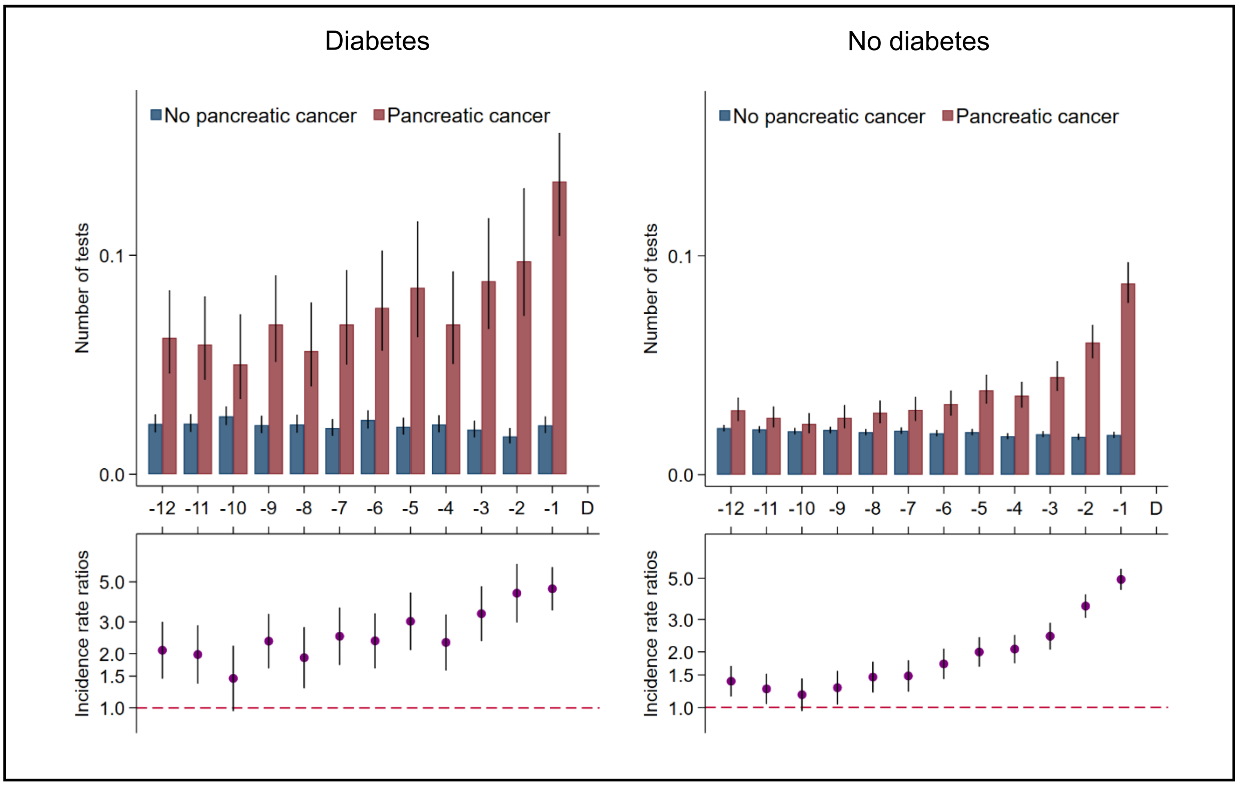

* Number of blood glucose tests in the 12 months prior to the pancreatic cancer diagnosis date and a corresponding index date assigned to references without pancreatic cancer. Number of tests are presented as crude rates of mean number of investigations per month (upper part) and incidence rate ratios (lower part) adjusted for age, marital status, ethnicity, educational level, household income and comorbidity. Black lines represent 95% confidence intervals
